# Adapting wine grape production to climate change through canopy architecture manipulation and irrigation in warm climates

**DOI:** 10.3389/fpls.2022.1015574

**Published:** 2022-10-03

**Authors:** Runze Yu, Nazareth Torres, Justin D. Tanner, Sean M. Kacur, Lauren E. Marigliano, Maria Zumkeller, Joseph Chris Gilmer, Gregory A. Gambetta, Sahap Kaan Kurtural

**Affiliations:** ^1^ Department of Viticulture and Enology, University of California, Davis, Davis, CA, United States; ^2^ Ecophysiologie et genomique fonctionnelle de la vigne (EGFV), Bordeaux Sciences Agro, Institut national de la recherche agronomique (INRAE), Université de Bordeaux, Institue des sciences de la vigne et du vin (ISVV), Villenave d’Ornon, France

**Keywords:** anthocyanins, climate change, irrigation, trellis systems, viticulture

## Abstract

Grape growing regions are facing constant warming of the growing season temperature as well as limitations on ground water pumping used for irrigating to overcome water deficits. Trellis systems are utilized to optimize grapevine production, physiology, and berry chemistry. This study aimed to compare 6 trellis systems with 3 levels of applied water amounts based on different replacements of crop evapotranspiration (ET_c_) in two consecutive seasons. The treatments included a vertical shoot position (VSP), two modified VSPs (VSP60 and VSP80), a single high wire (SH), a high quadrilateral (HQ), and a Guyot pruned VSP (GY) combined with 25%, 50%, and 100% ET_c_ water replacement. The SH had greater yields, whereas HQ was slower to reach full production potential. At harvest in both years, the accumulation of anthocyanin derivatives was enhanced in SH, whereas VSPs decreased them. As crown porosity increased (mostly VSPs), berry flavonol concentration and likewise molar % of quercetin in berries increased. Conversely, as leaf area increased, total flavonol concentration and molar % of quercetin decreased, indicating a preferential arrangement of leaf area along the canopy for overexposure of grape berry with VSP types. The irrigation treatments revealed linear trends for components of yield, where greater applied water resulted in larger berry size and likewise greater yield. 25% ET_c_ was able to increase berry anthocyanin and flavonol concentrations. Overall, this study evidenced the efficiency of trellis systems for optimizing production and berry composition in Californian climate, also, the feasibility of using flavonols as the indicator of canopy architecture.

## Introduction

Grapes are profitable fruit crop that are widely grown in the state of California, with an increasing need to accomplish cultural tasks mechanically (California Department of Food and Agriculture (CDFA), 2020; Kurtural and Fidelibus, 2021). However, there are many factors that are currently challenging the productivity, quality, and sustainability in wine grape vineyards, one being the increasingly significant global warming trend affecting California and the whole world (Venios et al., 2020; Rienth et al., 2021), where more frequent heat waves (Torres et al., 2021a) and continued warming of air temperature imposes great threats to vineyard yield, berry and wine composition (Gambetta and Kurtural, 2021).

Grape berry and wine quality are determined by the composition and concentration of secondary metabolites accumulated in berries. Flavonoids are the most abundant secondary metabolites and contribute to many quality-determining traits, including color, mouthfeel, and aging potential of wine (Poni et al., 2018). There are generally three classes of flavonoids in wine grapes, including anthocyanins, flavonols, and proanthocyanidins. Anthocyanins are responsible for grape berry and wine color, and they are sensitive to external environmental conditions when clusters are exposed to solar radiation and heat, with overexposure resulting in anthocyanin degradation (Torres et al., 2020; Torres et al., 2021a). On the other hand, flavonols tend to be positively related to solar radiation (Martínez-Lüscher et al., 2019a). Solar radiation, especially UV-B, can often up-regulate flavonols’ biosynthesis, resulting in more flavonols accumulated in berry skins. However, excessive solar radiation received and heat accumulated in California would accelerate the degradation of not only anthocyanins, but also flavonols, which will cause a decline in the antioxidant capacity of resultant wine and a possible reduction in wine aging potential (Torres et al., 2021a).

In viticulture, trellis system selection is a critical aspect grower needs to consider when establishing a vineyard. An ideal trellis can promote grapevines’ photosynthetic capacity through optimizing light interception by the grapevine canopy. Most importantly, a suitable trellis can optimize canopy microclimate by providing sufficient solar penetration into canopies since solar radiation is necessary to enhance the berry composition (Bavougian et al., 2012; Sanchez-Rodriguez and Spósito, 2020) without excessive exposure of clusters to direct sunlight to avoid flavonoid degradation (Martínez-Lüscher et al., 2020; Torres et al., 2021a). There is evidence that grape clusters over-exposed to solar radiation are prone to occur with some of the widely used trellis systems. For example, vertical shoot position (VSP), a traditional and commonly used trellis system in viticulture production, has been found to produce canopies with high porosity which increases vulnerability of clusters to over-exposure (Dry, 2009), causing overly enhanced maturity and considerable degradation in berry anthocyanins (Torres et al., 2021a). However, there is a lack of evaluations of the performance among various trellis systems in relation to the warming climate trends, and how their specific architectures contribute to variations in berry chemical profiles.

In warm climates such as California, viticulture relies on irrigation for maintaining production, and previous work in the area showed that the application of different amounts of crop evapotranspiration (ET_c_) can significantly modify polyphenolic and aromatic profiles in wine (Torres et al., 2022). Due to the increasingly frequent drought condition in many wine grape growing regions, recent studies have been focusing on the grapevine physiological and berry chemical responses towards specific levels of water deficits imposed by different ET_c_ replacements, where water deficits are affective in manipulating grapevine water status, leaf gas exchange, components of yield, and berry composition: often, more water deficits applied to the grapevines would diminish photosynthetic capacities, but promote berry maturity (*i.e.* sugar and flavonoid accumulation) (Torres et al., 2021d; Torres et al., 2021c; Torres et al., 2022). In some extremely drought conditions, however, severe water deficit might lower flavonoid concentration due to encouraged chemical degradation (Yu et al., 2020). Moreover, these effects resulted from different irrigation regimes can be modified by the canopy architecture as functions of trellis system since trellis systems can directly determine canopy sizes, hence resulting in different water demands from grapevines accordingly (Williams, 2000). On the other hand, over extraction of ground water to irrigate permanent crops have recently been questioned and legislation has been enacted in the state of California called the ‘Sustainable Groundwater Management Act’ (Kiparsky, 2016). As a result, in some regions such as Napa Valley of California, grape growers will only be allowed to irrigate 120 mm per year. However, there is a lack of information on how the existing vineyards will cope with this water limitation in terms of irrigation scheduling.

Therefore, the objectives of this study were to evaluate and compare 6 different trellis systems in combination with 3 irrigation strategies to understand the impact of trellis system and applied water amount on canopy architecture, grapevine physiology and berry composition. We hypothesized that traditional VSP systems would not be as efficient as the other trellis systems in terms of yield production and flavonoid accumulation, leading to greater berry flavonoid degradation and overall lower flavonoid concentrations.

## Materials and methods

### Vineyard site, plant materials, and weather conditions

The experiment was conducted in 2020 and 2021 on *Vitis vinifera* `Cabernet sauvignon´ (Clone 8) grapevines grafted on 3309C rootstock (*V. riparia* × *V. rupestris*). The vineyard for this study was located at the University of California Oakville Experimental Station in Oakville, Napa County, CA, USA and planted in 2016. Grapevines were spaced at 1.52 m × 2.13 m (vine × row). The rows had NE-SW orientation.

Weather data at this vineyard was obtained from the California Irrigation Management Information System (CIMIS) (station #77, Oakville, CA). The weather station was located approximately 100 m from the experimental vineyard block. Growing Degree Days (GDD) were used to assess the accumulated heat units at the experimental site, and calculated with the following equation (McMaster and Wilhelm, 1997):


1) $$\text{GDD}=\sum_{\text{Apri }1}^{\text{Harvest}}\left(\frac{\text{Daily maximum temperature}+\text{Daily minimum temperature}}{2}-10\right)$$

where negative values were not included in the accumulated GDD value, and the time period recorded for the calculation was from 1 April until harvest in each year.

### Experimental design

The study was conducted in a split-plot factorial design that utilized 2 separate sets of factors. The main factors of the experiment were 6 trellis systems randomly combined with 3 different water amounts applied at random to each row with 4 replications in each treatment, which consisted of seven vines. There were 72 treatment-replicates in total. The main plot factor (trellis systems) was applied to every row, and the sub-plot (applied water amounts) was applied randomly to 7 consecutive vines within each row so that 3 separate irrigation sub-plot factors were contained in every row within the vineyard block. The 5 middle vines in each treatment-replicate were used for on-site measurements as well as berry sampling.

### Trellis systems and applied water amounts

#### Trellis systems

**Figure 1:**
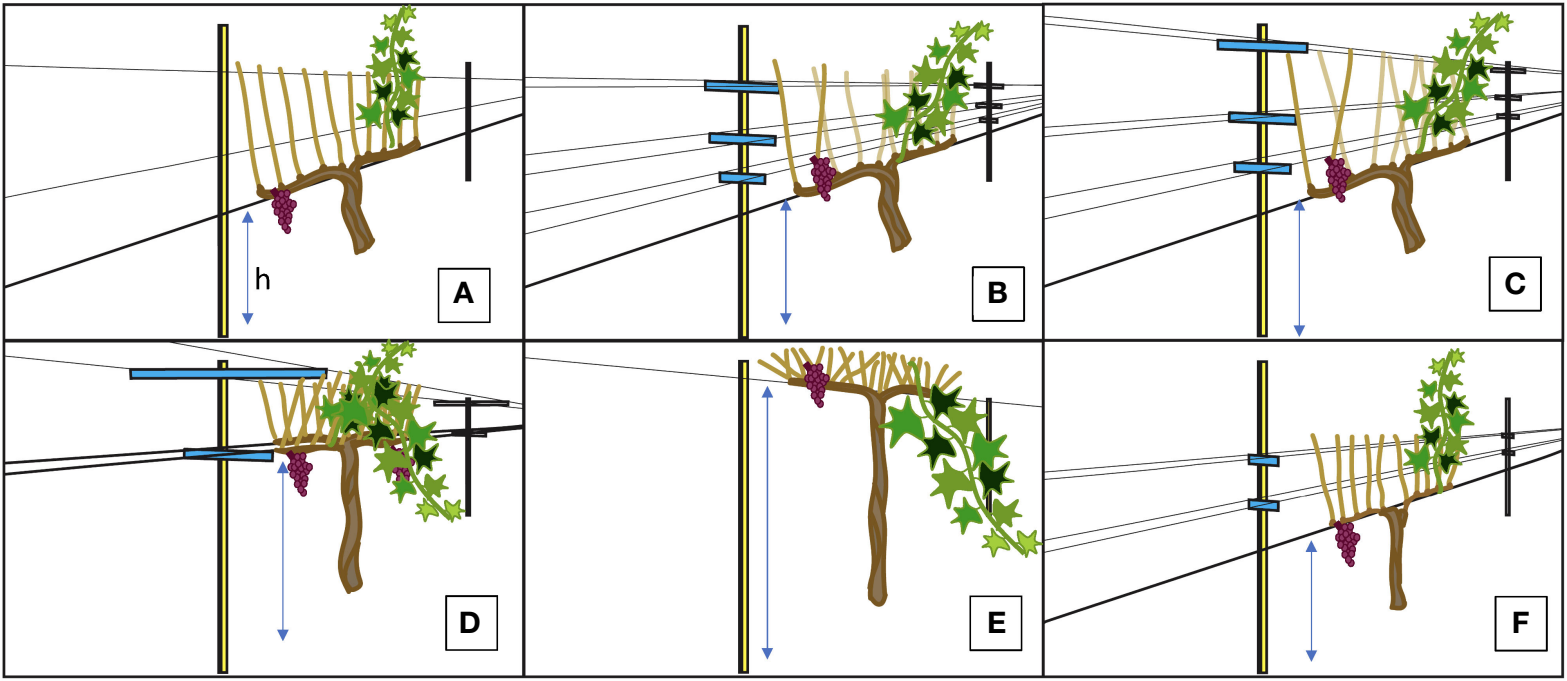
Illustrations for the Trellis Systems Established at the Oakville Experimental Vineyard: **(A)** Traditional Vertical Shoot Position (VSP); **(B)** Vertical Shoot Position 60^°^ (VSP60); **(C)** Vertical Shoot Position 80^°^ (VSP80); **(D)** High-Quadrilateral (HQ); **(E)** Single High Wire (SH); **(F)** Guyot-pruned Vertical Shoot Position (GY). “h” stands for the cordon height from the vineyard ground and the h for each trellis system was described in Materials and Methods.

6 trellising systems were used for the measurements in this experiment (**Figure 1**). The 6 trellis systems included a vertical shoot position (VSP, **Figure 1** **A**), 2 additional VSP designs that were modified with more opened canopies (with ~60°C and ~80°C shoot orientation: VSP60 and VSP80, **Figures 1** **B, C**, respectively), a VSP trellis cane-pruned with a Guyot method (GY, **Figure 1F**), a high quadrilateral trellis (HQ, **Figure 1D**), and a single high wire trellis (SH, **Figure 1E**). The cordon height (h) for 1A, 1B, 1C and 1F were 0.96 m above vineyard floor. The cordon height for 1D was 1.54 and for 1E it was 1.70 m above vineyard floor respectively

The canopy management was conducted based on the common local practices for these trellis designs for the traditional VSP, VSP60, and VSP80. The grapevines were spur-pruned to two buds per spur retaining approximately 30 spurs per plant. After bud break, the shoot numbers were corrected to approximately 25 shoots per vine for VSP types. The HQ grapevines were spur pruned to retain 60 spurs per plant and then shoot thinned to 50 shoots per vine. The SH vines were box-pruned mechanically to a spur height of approximately 10 cm, and 45% of the shoots were mechanically thinned at 40 cm shoot length as per common local practice to mimic manual shoot thinning operations (Terry and Kurtural, 2011; Kurtural et al., 2019). The GY vines were cane-pruned by hand to 2, 12-node canes with 2 renewal spurs at the head of each vine. There was no leaf removal or cluster removal conducted in either year.

#### Applied water amounts

The irrigation treatments applied to the grapevines were based on calculated ET_c_ by using the following equation:


2) $${\text{ET}}_{\text{c}}={\text{ET}}_{\text{o}}\times {\text{K}}_{\text{c}}$$

where ET_o_ was the reference evapotranspiration and K_c_ is the crop coefficient. ET_o_ was measured from the CIMIS station weekly throughout both seasons, and K_c_ was assessed by using the shade cast method previously described by Williams and Ayars (2005). Briefly, three neighboring VSP trellised rows were irrigated to 100% of ET_o_ to create unstressed grapevines. Shade cast on to the berm and row middles were measured weekly then to calculate the K_c_. Irrigation was initiated when the general grapevine stem water potential for the field fell below – 1.0 MPa (28 May 2020 and 10 June 2021). The applied water amounts used in this study were to replace 100% crop evapotranspiration (ET_c_), 50% ET_c_ and 25% ET_c_. These treatments were applied by varying the emitter numbers per vine with irrigation duration determined based on 100% ET_c_ treatment. NETAFIM™ pressure compensating on-line button drippers were installed to apply different rates of irrigation: 2 drippers with a rate of 4 L/h at each vine to simulate 100% ET_c_ replacement, 2 drippers with a flow rate of 2 L/h at each vine to simulate 50% ET_c_ replacement, and 2 drippers with a flow rate of 1 L/h to simulate 25% ET_c_. In total, 100% ET_c_ treated grapevines received 308 mm and 246 mm of water in 2020 and 2021, respectively.

### Leaf gas exchange, leaf area index, and yield component assessment

#### Leaf gas exchange

At mid-day (between 12:00 – 14:00 h), leaf gas exchange measurements were taken bi-weekly in both seasons to assess leaf photosynthetic activities as well as plant water status by using a portable infrared gas analyzer CIRAS-3 (PP Systems, Amesbury, MA, USA). Each time, three different fully sun-exposed leaves were selected from the main shoot axis on the middle three grapevines in each treatment-replicate. In both years, the measurements were taken when sunlight condition were at photosynthesis saturation levels, where the average photosynthetic active radiation (PAR) was approximately at 1708.43 ± 282.81 μmol mol^-1^ (mean ± one standard deviation from the mean) in 2020 and 1764.85 ± 287.84 μmol mol^-1^ (mean ± one standard deviation from the mean) in 2021. CIRAS-3 was set to a relative humidity at 40% and a reference CO_2_ concentration at 400 μmol mol^−1^. From the measurement, leaf net carbon assimilation (*A*_net_) and stomatal conductance (*g_s_*) were assessed directly. Intrinsic water use efficiency (WUE_i_) was calculated as the ratio between *g*_s_ to *A*_net_.

#### Canopy microclimate and leaf area

Canopy microclimate was assessed using digital photography as previously reported (Martínez-Lüscher et al., 2019a). Crown porosity (% of gaps in the canopy) and leaf area index (LAI) was assessed with a smartphone application VitiCanopy on an iOS operating system (Apple Inc., Cupertino CA, USA) (de Bei et al., 2016). The settings used for this vineyard site were described previously (Yu et al., 2021b). Total leaf areas were calculated based on the LAI multiplied by the unit ground area for each vine (3.24 m^2^).

#### Yield components

Clusters were harvested by hand at approximately 23 - 25 °CBrix, and all clusters in each treatment-replicate were harvested, counted, and weighed on a single harvest day each season (14 September 2020, 6 September 2021). Yield components were assessed or calculated for cluster number per vine, cluster weight, berry fresh weight, leaf area to fruit ratio, and yield per vine.

### Berry sampling and berry quality assessment

10 berries were randomly sampled from each of the five central vines for a total of 50 berries. Berry samplings took place at harvest in both seasons. The 50 berries were divided into two subsets of 30 berries and 20 berries. The 30-berry set was used for berry weight and berry composition analysis. Berry must total soluble solids (TSS) was recorded in the unit of °CBrix with a digital refractometer (Atago PR-32, Bellevue, WA, USA). Measurements of the berry must pH and titratable acidity (TA) were determined with an autotitrator (862 Compact TitroSampler, Metrohm, Switzerland) and were recorded as g L^-1^ of tartaric acid at the titration end point of pH 8.2 (Ough and Amerine, 1988).

### Sample preparation for determination of skin flavonoids

The second subset of 20 berries was used for the determination of skin flavonoids from each individual treatment-replicate. Skins were manually removed from the subset of 20 berries and subsequently lyophilized (Centrivap Benchtop Centrifugal Vacuum Concentrator 7810014 equipped with Centrivap -105°C Cold Trap 7385020, Labconco, Kansas City, MO, USA). After lyophilization, dry skin weights were recorded and then, the dried skins were ground into fine powder with a mixing mill (MM400, Retsch, Mammelzen, Germany). 50 mg of the freeze-dried berry skin powder were collected, and the skin flavonoids were extracted with 1 mL of methanol:water:7M hydrochloric acid (70:29:1, V:V:V) to simultaneously determine flavonol and anthocyanin concentration and profile as previously described by Martínez-Lüscher et al. (2019a). The extracts were stored overnight in a refrigerator at 4°C. In the next day, the extracts were centrifuged at 30,000 *g* for 15 minutes, and the supernatants were separated from the solids and transferred into HPLC vials after being filtered by PTFE membrane filters (diameter: 13 mm, pore size: 0.45 μm, VWR, Seattle, WA, USA). Then, the samples were injected into HPLC for chromatographic analysis.

### Determination of berry skin flavonoids

Anthocyanin and flavonol concentrations (expressed in the unit of mg per g of berry fresh weight) in berry skin tissues were analyzed with a reversed-phase HPLC (Model 1260, Agilent, Santa Clara, CA, USA) with the use of two mobile phases: (A) 5.5% formic acid in water and (B) 5.5% formic acid in acetonitrile. The specific method used for this study required a C18 reversed-phase HPLC column for the analysis (LiChrosphere 100 RP-18, 4 × 520 mm^2^, 5 mm particle size, Agilent Technologies, Santa Clara, CA, United States). The flow rate of the mobile phase was 0.5 mL min^-1^ and the flow gradient started with 91.5% A with 8.5% B, 87% A with 13% B at 25 min, 82% A with 18% B at 35 min, 62% A with 38% B at 70 min, 50% A with 50% B at 70.01 min, 30% A with 70% B at 75 min, 91.5% A with 8.5% B from 75.01 min to 90 min. The column temperature was maintained at 25°C on both left and right sides of the column. All chromatographic solvents were of high-performance liquid chromatography (HPLC) grade, including acetonitrile, methanol, hydrochloric acid, formic acid. These solvents were purchased from Thermo-Fisher Scientific (Santa Clara, CA, USA). Detection of flavonols and anthocyanins was recorded by the diode array detector (DAD) at 365 and 520 nm, respectively. Investigated anthocyanin derivatives included di-hydroxylated forms: cyanidin and peonidin, and tri-hydroxylated forms: delphinidin, petunidin, and malvidin; investigated flavonols included a mono-hydroxylated form: kaempferol, di-hydroxylated forms: quercetin and isorhamnetin, and tri-hydroxylated forms: myricetin, laricitin, and syrigintin.

Post-run chromatographic analysis was conducted with Agilent OpenLAB software (Chemstation edition, version A.02.10) and identification of individual anthocyanins and flavonols was made by comparison of the commercial standard retention times found in the literature (Martínez-Lüscher et al., 2019a). Malvidin 3-*O*-glucoside used for anthocyanin identification was purchased from Extrasynthese (Genay, France). Myricetin-3-*O*-glucuronide, myricetin 3-*O*-glucoside, quercetin 3-*O*-glucuronide, quercetin 3-*O*-galactoside, quercetin 3-*O*-glucoside, kaempferol 3-*O*-glucoside, isorhamnetin 3-*O*-glucoside, and syringetin 3-*O*-glucoside used for flavonol identification were purchased from Sigma-Aldrich (St. Louis, MO, United States). Flavonol molar abundant (molar %) was calculated as the percentage of specific flavonol derivatives’ concentration over total flavonols’ concentration.

### Statistical analysis

The statistical analysis for the experiment was performed using MIXED procedure of SAS (v 9.4. SAS Institute, Cary, NC, USA). All the datasets were first checked for normal distribution using a Shapiro-Wilkinson test before running the two-way MIXED procedure. A Tukey’s HSD *post-hoc* test was performed to analyze the degree of significance among the various measurements. The levels of significance ≤ 0.10 were the results that were considered for the Tukey’s *post hoc* tests. Season-long measurements of leaf gas exchange variables were analyzed for each year *via* three-way Analysis of Variance using the MIXED procedure of SAS using REPEATED option for measurement dates. A regression analyses was performed between variables of interest and, *p* values were acquired to present the significances of the linear fittings, as well as the regression coefficient (as R^2^).

## Results

### Weather at the experimental site

Both seasons were considerably arid as the experimental site only received 233.9 mm and 276.9 mm of precipitation from the previous dormant season until harvest in 2020 and 2021, respectively (**Table 1**). During the growing seasons, from April to September, the site received 23.2% of the total precipitation in 2020 (54 mm) and only 2.1% in 2021 (5.9 mm). In addition, there was minimal precipitation during data collection of this study from June to September, where only 2 mm and 1.2 mm of precipitation were received in 2020 and 2021. As for the air temperature during the growing seasons, the average maximum air temperature was slightly higher in July, August, and September in 2020 compared to 2021, but lower in March and April. The average minimum air temperature was constantly higher in 2020 compared to 2021 from March until harvest in September, except July. Similarly, the average air temperature was generally higher in 2020 than 2021 except both Julys which had the same average air temperature. As for GDD accumulation (as calculated until harvest), the two seasons were slightly different. In 2020, there was 1525.4°C GDD accumulated when the berries reached 23.9°Brix on average; in 2021, there was 1292.3°C GDD accumulated when the berries reached 22.6°Brix on average. Thus, 2020 was a slightly drier and hotter season than 2021.

**Table 1:** Weather information at the experimental site as obtained from california irrigation management information system (cimis) station located in oakville (#77, Oakville, Napa County)^^a^^.

Month-Year	Precipitation (mm)	Average Maximum Air Temperature (°C)	Average Minimum Air Temperature (°C)	Average Air Temperature (°C)	Growing Degree Days (°C)
Oct-19	0.2	26.6	4.9	15.4	–
Nov-19	24.4	20.8	3.3	11	–
Dec-19	66	14.3	5.7	9.5	–
Jan-20	58.5	15.4	3.5	8.8	–
Feb-20	1	20.6	3.7	11.4	–
Mar-20	29.8	17.6	4.4	10.7	–
Apr-20	25.9	23	7.1	14.6	154.2
May-20	26.1	26.2	8.8	17.4	385.05
Jun-20	0.2	29.5	10.4	19.7	683.8
Jul-20	0.2	30.2	10.1	19.2	997.35
Aug-20	1.6	31.8	12.3	21.1	1359.15
Sep-20	0	31.4	11.1	20	1525.35
Oct-20	0.3	29.7	8.1	17.4	–
Nov-20	31.9	19.8	2.2	10.2	–
Dec-20	46.4	17.1	2	8.5	–
Jan-21	97.5	15.7	3.6	9	–
Feb-21	35.3	18	3.3	10.5	–
Mar-21	59.6	18.5	2.6	10.3	–
Apr-21	4.3	23.5	4.3	13.1	116.55
May-21	0.4	27.7	7.2	17.3	347.6
Jun-21	0.3	28.7	9.3	18.8	618.1
Jul-21	0.2	29.5	10.8	19.2	932.6
Aug-21	0.2	29.7	10.1	19	1239.5
Sep-21	0.5	30	8.7	18.6	1292.25

a Growing degree days were calculated from 1 April to harvest in each year.

### Canopy microclimate

**Figure 2:**
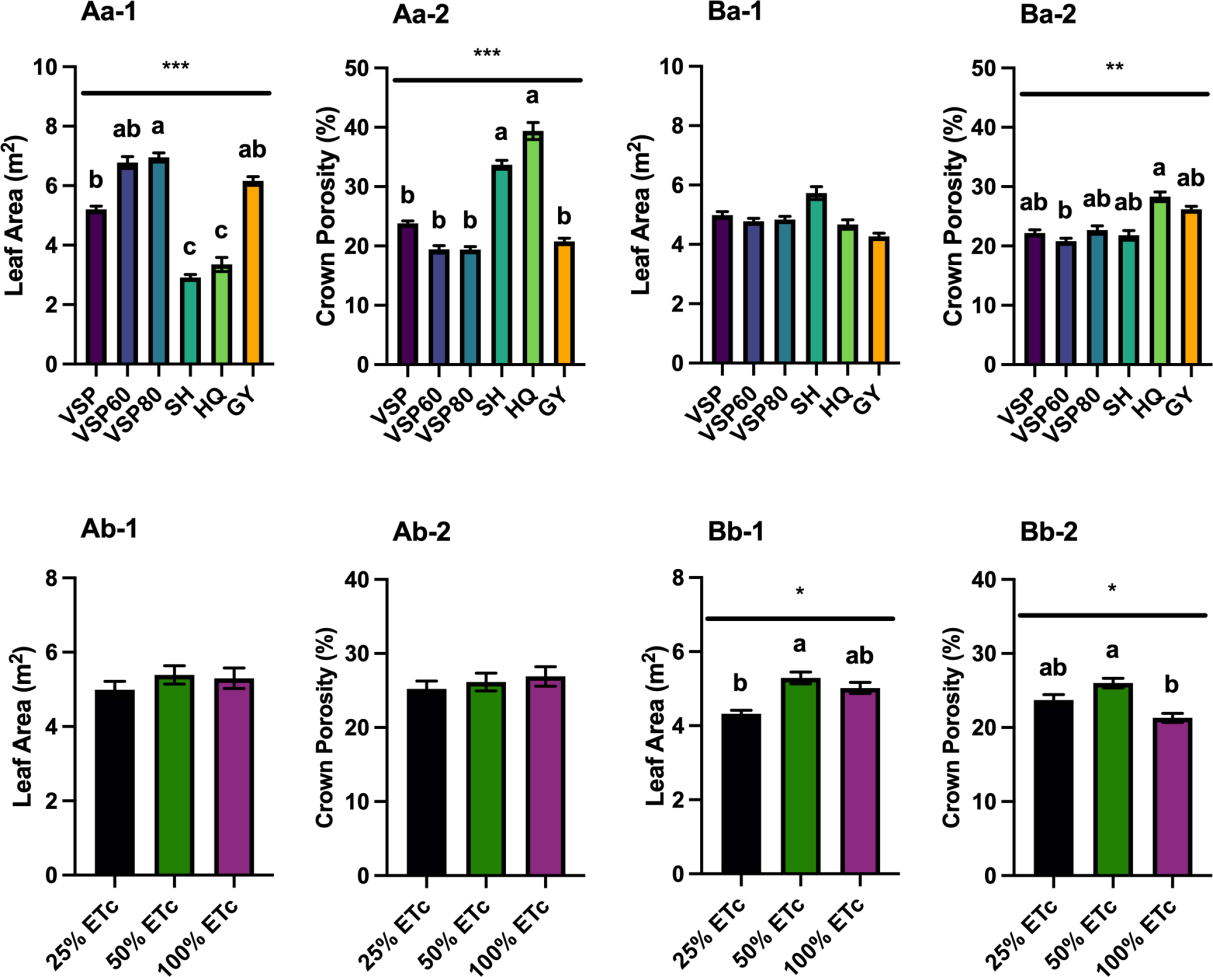
Canopy Architecture as Affected by Trellis Systems and Applied Water Amounts of ‘Cabernet Sauvignon’ in Oakville, CA, USA in **(A)** 2020 and **(B)** 2021; **(a)** the main effect of trellis systems, **(b)** the main effect of applied water amounts; (1) leaf area, (2) crown porosity. Error bars represent one standard deviation from the mean, letters represent ranking after Tukey’s *post hoc* analyses. Asterisks represents significant levels *p*, ‘***’*p*< 0.001, ‘**’*p* < 0.01, ‘*’*p* < 0.05. VSP, vertical shoot position; VSP60, vertical shoot position 60°; VSP80, vertical shoot position 80°; SH, single hire wire; HQ, High-Quadrilateral; GY, guyot-pruned vertical shoot position; ET_c_, crop evapotranspiration.

LAI and crown porosity were assessed in both seasons, and leaf areas were calculated based on the unit ground area and LAI (**Figure 2**). In 2020, VSP80 had the most leaf area among the six trellis systems, VSP60 and GY had similar leaf areas, followed by VSP (**Figure 2** **Aa-1**). SH and HQ had the lowest leaf areas as the canopies in these two trellises still had gaps. This was also confirmed with the fact that SH and HQ had the highest crown porosities among the six trellis systems (**Figure 2** **Aa-2**). The other trellis systems had similar lower crown porosities than SH and HQ. There was no difference in canopy architecture among the three irrigation regimes in the first season (**Figures 2** **Ab-1, Ab-2**).

In 2021, all the trellis systems had similar leaf areas (**Figure 2** **Ba-1**). HQ had higher crown porosity than VSP60, but the other trellis systems had similar crown porosities to either HQ or VSP60 (**Figure 2** **Ba-2**). These effects were not modified by the irrigation treatments and no significant interactions between factors were found. For applied water amounts, 50% ET_c_ had higher leaf area than 25% ET_c_, but there was no difference between 100% ET_c_ with either 25% or 50% (**Figure 2** **Bb-1**). However, 50% ET_c_ still had the highest crown porosity compared to 100% ET_c_, and 25% ET_c_ did not show any difference with the other two irrigation treatments (**Figure 2** **Bb-2**).

### Grapevine leaf gas exchange

**Figure 3:**
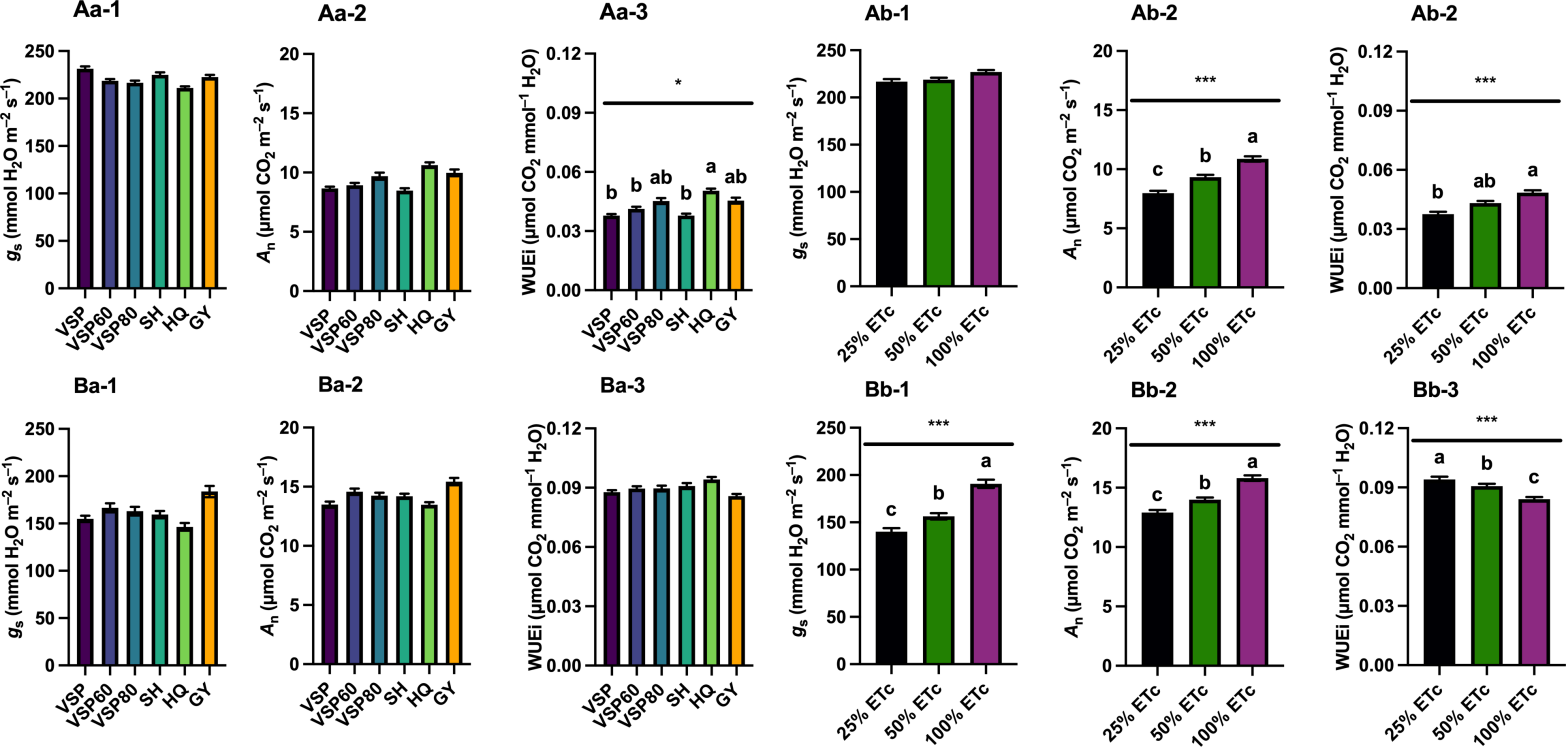
Season-long Leaf Gas Exchange Integrals as Affected by Trellis Systems and Applied Water Amounts of ‘Cabernet Sauvignon’ in Oakville, CA, USA in **(A)** 2020 and **(B)** 2021; **(a)** the main effect of trellis systems, **(b)** the main effect of applied water amounts; (1) stomatal conductance (*g*_s_), (2) net carbon assimilation rate (*A*_n_), (3) intrinsic water use efficiency (WUE_i_). Error bars represent one standard deviation from the mean, letters represent ranking after Tukey’s *post hoc* analyses. Asterisks represents significant levels *p*, ‘***’*p*< 0.001, ‘*’*p*< 0.05. VSP, vertical shoot position; VSP60, vertical shoot position 60°; VSP80, vertical shoot position 80°; SH, single hire wire; HQ, High-Quadrilateral; GY, guyot-pruned vertical shoot position; ET_c_, crop evapotranspiration.

Grapevine leaf gas exchange was monitored throughout both seasons, and their integrals were calculated to represent the season-long plant response of grapevines for net carbon assimilation rate (*A*_n_), stomatal conductance (*g*_s_), and intrinsic water use efficiency (WUE_i_) (**Figure 3**). In 2020, there were no differences in *g*_s_ and *A*_n_ among the six trellis systems (**Figures 3** **Aa-1, Aa-2**). However, HQ had the highest WUE_i_, whereas VSP, VSP60, and SH had lower WUE_i_ (**Figure 3** **Aa-3**). Regarding the irrigation treatments, there was no difference in *g*_s_ integrals (**Figure 3** **Ab-1**). However, a linear response to water amounts were observed for *A*_n_ and WUE_i_, with 100% ET_c_ having the highest values of both gas exchange variable monitored (**Figures 3** **Ab-2, Ab-3**).

In 2021, there were no differences in *g*_s_, *A*_n_, and WUE_i_ among the six trellis systems (**Figures 3** **Ba-1, Ba-2, Ba-3**). Nevertheless, a linear response to water amounts was recorded, with 100% ET_c_ showing the highest *A*_n_ and *g*_s_, followed by 50% ET_c_, and 25% ET_c_ (**Figure 3** **Bb-1, Bb-2**) which accounted for a higher WUE_i_ in 25% ET_c_ with 50% treatments compared with 100% ET_c_ (**Figure 3** **Bb-3**).

**Figure 4:**
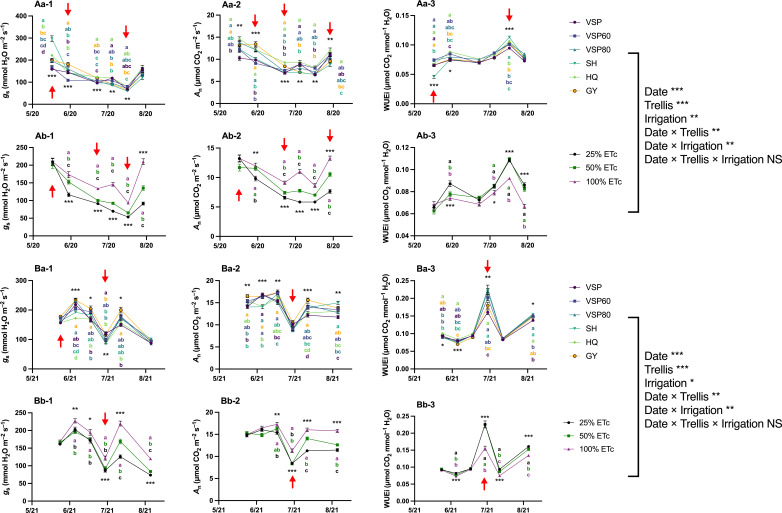
Progression of Leaf Gas Exchange as Affected by Trellis Systems and Applied Water Amounts of ‘Cabernet Sauvignon’ in Oakville, CA, USA in **(A)** 2020 and **(B)** 2021; **(a)** the main effect of trellis systems, **(b)** the main effect of applied water amounts; (1) stomatal conductance (*g*_s_), (2) net carbon assimilation rate (*A*_n_), (3) intrinsic water use efficiency (WUE_i_). Error bars represent one standard deviation from the mean, letters represent ranking after Tukey’s *post hoc* analyses. Asterisks represents significant levels *p*, ‘***’*p*< 0.001, ‘**’*p*< 0.01, ‘*’*p*< 0.05. Arrows above individual dates indicate statistical difference between starting date and the indicated date. VSP, vertical shoot position; VSP60, vertical shoot position 60°; VSP80, vertical shoot position 80°; SH, single hire wire; HQ, High-Quadrilateral; GY, guyot-pruned vertical shoot position; ET_c_, crop evapotranspiration. ns, not signigficant.

The analysis of the gas exchange recorded at each measurement day indicated that in 2020, despite starting with the highest *g*_s_, SH had lower *g*_s_ over the season (**Figure 4** **Aa-1**). Contrarily, HQ trellis system showed higher *g*_s_ in July and August which was connected with higher *A*_n_ over the season (**Figure 4** **Aa-2**). On the other hand, GY and VSP80 systems enhanced *A*_n_ during some periods over the season. Regarding WUE_i_, VSP60 and HQ had the highest values while SH decreased it in the early season and increased it in early August (**Figure 4** **Aa-3**). However, all these differences tended to diminish at the end of the season. For irrigation treatments, a constant effect of water amount was observed with 100% ET_c_ increasing *g*_s_ and *A*_n_ and decreasing WUE_i_ (**Figures 4** **Ab-1, Ab-2, Ab-3**).

In 2021, GY and VSP60 showed higher *g*_s_ and *A*_n_ values in general (**Figures 4Ba-1, Ba-2**). HQ showed lower *g*_s_ values and VSP had lower *A*_n_ values compared to the other trellis systems throughout the season. HQ increased WUE_i_ throughout the whole season (**Figure 4Ba-3**). Although GY had higher WUE_i_ in the early season, it showed constantly lower WUE_i_ values after 23 June 2021. Besides GY, VSP showed lower WUE_i_ in July and August. A similar effect of irrigation treatments was observed over the second season, with a linear response for increased *g*_s_ and *A*_n_ and decreased WUE_i_ when the irrigation water amount was increased (**Figures 4** **Bb-1, Bb-2, Bb-3**).

### Yield components and berry quality parameters

Yield components and berry quality parameters were assessed at harvest in both seasons (**Table 2**). SH and HQ had the smallest berries among the six trellis systems in the two seasons. In 2020, SH and VSP increased the cluster number, while VSP80 and GY decreased it whereas, in 2021, SH and HQ accounted for increased the cluster number. VSP, VSP60, VSP80, and GY increased the cluster weight compared to SH in 2020. In 2021, SH showed the lowest cluster weight and skin weight. Regarding yield, differences were only significant in 2020 where SH enhanced vine yield compared to the other trellis systems. On the other hand, 100% ET_c_ enhanced berry weight, cluster weight, and yield over the two seasons with no difference on leaf area to fruit ratio. Regarding berry quality parameters, SH had the highest TSS among and the lowest pH in 2020, whereas in 2021, VSPs and GY enhanced the TSS and the pH. Results also showed that irrigation treatments had little effect on the berry quality parameters over the two seasons with only TSS being increased in the 25% ET_c_ treatment in the harvest of 2020.

**Table 2:** Effects of trellis systems and applied water amounts on yield components and berry composition of ‘Cabernet Sauvignon’ in Oakville, CA in 2020 and 2021^^a^,^b^^.

		*Trellis*							*Irrigation*				Trellis×Irrigation
		VSP	VSP60	VSP80	SH	HQ	GY	*p value*	25%ET_c_	50%ET_c_	100%ET_c_	*p value*	
**2020**	**Berry Weight (g)**	0.97 **a**	1.00 **a**	1.01 **a**	0.86 **b**	0.87 **b**	0.97 **a**	**	0.83 **c**	0.96 **b**	1.05 **a**	***	ns
	**Cluster No.**	62.97 **a**	32.39 **bc**	30.36 **c**	62.97 **a**	38.11 **b**	27.67 **c**	***	35.96	38.04	38.07	ns	ns
	**Cluster Weight (g)**	132.76 **a**	133.40 **a**	120.48 **a**	74.17 **c**	97.24 **b**	138.97 **a**	***	100.00 **b**	118.39 **a**	130.12 **a**	***	ns
	**Skin Weight (mg)**	30.09 **b**	30.87 **b**	34.52 **b**	44.90 **a**	32.73 **b**	32.27 **b**	***	3491	35.97	31.81	ns	ns
	**Yield (kg vine^-1^)**	4.27 **ab**	4.34 **ab**	3.56 **b**	4.71 **a**	3.56 **b**	3.87 **b**	*	3.39 **b**	4.19 **a**	4.58 **a**	**	ns
	**Leaf Area: Fruit (m^2^ kg^-1^)**	1.09	1.03	1.07	1.45	1.10	1.12	ns	1.19	1.13	1.12	ns	ns
	**TSS (°Brix)**	23.5 **b**	23.7 **b**	23.7 **b**	24.6 **a**	24.1 **ab**	24.2 **ab**	*	24.8 **a**	24.1 **b**	22.9 **c**	***	ns
	**pH**	3.47 **a**	3.49 **a**	3.46 **ab**	3.40 **c**	3.42 **bc**	3.48 **a**	**	3.47	3.45	3.44	ns	ns
	**TA (g L^-1^)**	7.74	7.36	7.69	7.53	7.78	7.71	ns	7.50	7.70	7.80	ns	ns
**2021**	**Berry Weight (g)**	1.00 **ab**	1.03 **a**	1.03 **a**	0.88 **ab**	0.83 **b**	1.03 **a**	**	0.88 **b**	0.94 **b**	1.07 **a**	***	ns
	**Cluster No.**	45.51 **b**	48.86 **b**	44.50 **b**	88.28 **a**	82.61 **a**	39.86 **b**	***	55.94	56.97	62.04	ns	ns
	**Cluster Weight (g)**	148.72 **ab**	153.96 **a**	149.24 **ab**	96.97 **b**	126.54 **ab**	168.59 **a**	**	121.78 **b**	156.19 **a**	144.04 **ab**	.	ns
	**Skin Weight (mg)**	64.82 **abc**	71.66 **ab**	66.84 **abc**	53.55 **c**	57.00 **bc**	73.86 **a**	**	60.40	64.11	69.36	ns	ns
	**Yield (kg vine^-1^)**	6.83	7.47	6.56	8.20	10.47	6.71	ns	6.13 **b**	8.68 **a**	8.21 **a**	.	ns
	**Leaf Area: Fruit (m^2^ kg^-1^)**	0.74	0.66	0.76	0.70	0.56	0.65	ns	0.72	0.70	0.62	ns	ns
	**TSS (°Brix)**	23.1 **a**	23.2 **a**	23.3 **a**	21.7 **b**	21.9 **b**	22.7 **ab**	*	22.6	22.5	22.9	ns	ns
	**pH**	3.62 **a**	3.59 **ab**	3.57 **ab**	3.55 **b**	3.53 **b**	3.58 **ab**	.	3.59	3.56	3.57	ns	ns
	**TA (g L^-1^)**	5.98	5.96	5.89	5.63	5.71	8.43	ns	6.81	5.82	6.18	ns	ns

a Analysis of variance (p value indicated) Letters within columns indicate significant mean separation according to Tukey’s test at where “.”: p value< 0.1; where “*”: p value< 0.05; “**”: p value< 0.001, “***”: p value< 0.0001.

b VSP, vertical shoot positioned; VSP 60, vertical shoot positioned 60°; VSP 80, vertical shoot positioned 80°; SH, single high wire; HQ, high quadrilateral; GY, guyot; TSS, total soluble solids; TA, titratable acidity; ET_c_, crop evapotranspiration; ns, not significant.

### Berry skin anthocyanins and flavonols

Berry skin anthocyanins were assessed in both seasons at harvest (**Table 3**). Different trellis systems affected not only the total anthocyanin concentration but also modified the anthocyanin composition, leading to modifications in the profile stability. In both seasons, SH had the highest concentrations in all the anthocyanin derivatives besides di- and tri-hydroxylated anthocyanins among the six trellis systems. In 2021, HQ also notably increased most of the anthocyanin derivatives, tri-hydroxylated, di-hydroxylated, and total anthocyanins compared to the VSP trellis systems. On the other hand, VSP trellis systems tended to decrease the anthocyanin concentrations. Regarding the irrigation treatments, 25% ET_c_ generally showed the higher concentrations in petunidins, di- and tri-hydroxylated anthocyanins, and total anthocyanins in 2020 compared to 100% ET_c_. In 2021, 25% ET_c_ increase most of the anthocyanin concentration in berries. 50% ET_c_ performed similarly in 2021 and showed higher concentrations in malvidins, tri-hydroxylated anthocyanins, and total anthocyanins.

**Table 3:** Effects of trellis systems and applied water amounts on berry skin anthocyanins of ‘cabernet sauvignon’ in Oakville, CA, USA in 2020 and 2021^a^ ^,^ ^b^ ^,^ ^c^.

		*Trellis*							*Irrigation*				Trellis×Irrigation
		VSP	VSP60	VSP80	SH	HQ	GY	*p value*	25%ET_c_	50%ET_c_	100%ET_c_	*p value*	
**2020**	**Cya**	0.02 **c**	0.03 **abc**	0.02 **bc**	0.04 **ab**	0.04 **a**	0.03 **abc**	*	0.03	0.03	0.03	ns	ns
	**Peo**	0.10 **b**	0.10 **b**	0.11 **b**	0.14 **a**	0.14 **a**	0.11 **b**	**	0.11	0.12	0.11	ns	ns
	**Di-OH**	0.12 **b**	0.13 **b**	0.13 **b**	0.18 **a**	0.17 **a**	0.13 **b**	*	0.14	0.15	0.14	ns	ns
	**Del**	0.15 **c**	0.18 **bc**	0.18 **bc**	0.31 **a**	0.22 **b**	0.17 **bc**	***	0.21	0.21	0.18	ns	ns
	**Pet**	0.12 **c**	0.14 **bc**	0.14 **bc**	0.23 **a**	0.17 **b**	0.13 **bc**	***	0.17 **a**	0.16 **ab**	0.14 **b**	**	ns
	**Mal**	0.96 **b**	0.95 **b**	1.01 **b**	1.36 **a**	1.05 **b**	0.94 **b**	***	1.20 **a**	1.03 **b**	0.92 **b**	***	ns
	**Tri-OH**	1.23 **b**	1.28 **b**	1.33 **b**	1.89 **a**	1.44 **b**	1.25 **b**	***	1.58 **a**	1.40 **ab**	1.23 **b**	***	ns
	**Total**	1.35 **b**	1.41 **b**	1.46 **b**	2.06 **a**	1.61 **b**	1.39 **b**	***	1.72 **a**	1.55 **ab**	1.38 **b**	**	ns
**2021**	**Cya**	0.02 **c**	0.02 **bc**	0.02 **c**	0.04 **a**	0.03 **ab**	0.02 **c**	***	0.03	0.03	0.02	ns	ns
	**Peo**	0.11 **b**	0.11 **b**	0.11 **b**	0.17 **a**	0.15 **a**	0.10 **b**	***	0.14 **a**	0.13 **ab**	1.74 **b**	ns	ns
	**Di-OH**	0.13 **b**	0.14 **b**	0.13 **b**	0.21 **a**	0.19 **a**	0.12 **b**	***	0.16 **a**	0.16 **ab**	0.13 **b**	*	ns
	**Del**	0.20 **b**	0.23 **b**	0.22 **b**	0.45 **a**	0.39 **a**	0.19 **b**	***	4.69	4.46	3.58	ns	ns
	**Pet**	0.16 **b**	0.19 **b**	0.19 **b**	0.34 **a**	0.31 **a**	0.16 **b**	***	0.26 **a**	0.24 **ab**	0.19 **b**	***	ns
	**Mal**	1.58 **b**	1.67 **b**	1.69 **b**	2.10 **a**	2.13 **a**	1.55 **b**	***	1.93 **a**	1.84 **a**	1.58 **b**	***	ns
	**Tri-OH**	1.95 **b**	2.09 **b**	2.11 **b**	2.89 **a**	2.83 **a**	1.90 **b**	***	2.51 **a**	2.38 **a**	1.99 **b**	***	ns
	**Total**	2.08 **b**	2.22 **b**	2.24 **b**	3.09 **a**	3.01 **a**	2.02 **b**	***	2.68 **a**	2.54 **a**	2.12 **b**	***	ns

a Analysis of variance to compare data (p value indicated); Letters within columns indicate significant mean separation according to Tukey’s HSD test at p value< 0.1, where “.”; p value< 0.05, where “*”: p value< 0.05; “**”: p value< 0.001, “***”: p value< 0.0001.

b VSP, vertical shoot positioned; VSP 60, vertical shoot positioned 60°; VSP 80, vertical shoot positioned 80°; SH, single high wire; HQ, high quadrilateral; Del, delphinidins; Cya, cyanidins; Pet, petunidins; Peo, peonidins; Mal, malvidins; Tri-OH, tri-hydroxylated anthocyanins; Di-OH, di-hydroxylated anthocyanins; ET_c_, crop evapotranspiration; ns, not significant.

c All compounds were expressed in the unit of mg per g of berry fresh weight.

In parallel with anthocyanin assessments, berry skin flavonols were measured at harvest in both seasons (**Table 4**). In 2020, SH showed the highest concentration in myricetins. SH and HQ showed the highest concentrations in quercetins, isorhamnetins and kaempferols in both seasons. SH and HQ also showed the highest concentration in tri- and di-hydroxylated as well as total flavonols in both seasons. In 2020, there were no differences among the six trellis systems in laricetins and syringetins. While in 2021, VSPs enhanced syringetin concentration. Regarding applied water amounts, little effects of irrigation treatments were shown in 2020. However, in 2021, 25% ET_c_ increased most of the flavonol derivatives except laricetins and syringetins compared to the other two treatments.

**Table 4:** Effects of trellis systems and applied water amounts on berry skin flavonols of ‘cabernet sauvignon’ in Oakville, CA, USA in 2020 and 2021^a^ ^,^ ^b^ ^,^ ^c^.

		*Trellis*							*Irrigation*				Trellis×Irrigation
		VSP	VSP60	VSP80	SH	HQ	GY	*p value*	25%ET_c_	50%ET_c_	100%ET_c_	*p value*	
	**Kae**	0.46 **bc**	0.36 **c**	0.42 **bc**	0.53 **ab**	0.66 **a**	0.55 **ab**	**	0.55	0.49	0.45	ns	ns
**2020**	**Que**	4.27 **b**	3.94 **b**	4.29 **b**	5.76 **a**	6.47 **a**	4.55 **b**	***	5.18	4.82	4.64	ns	ns
	**Iso**	0.47 **b**	0.42 **b**	0.48 **b**	0.52 **ab**	0.66 **a**	0.51 **ab**	**	0.53	0.51	0.48	ns	ns
	**Di-OH**	4.74 **b**	4.36 **b**	4.77 **b**	6.29 **a**	7.12 **a**	5.06 **b**	***	5.71	5.33	5.13	ns	ns
	**Myr**	2.40 **b**	2.40 **b**	2.57 **b**	3.46 **a**	3.17 **b**	2.59 **b**	**	2.84	2.81	2.65	ns	ns
	**Lar**	0.41	0.41	0.46	0.45	0.47	0.45	ns	0.46	0.45	0.41	ns	ns
	**Syr**	0.69	0.64	0.73	0.79	0.71	0.74	ns	0.79	0.72	0.63	ns	ns
	**Tri-OH**	3.49 **c**	3.46 **c**	3.76 **bc**	4.70 **a**	4.35 **ab**	3.78 **bc**	*	4.10	3.98	3.69	ns	ns
	**Total**	8.69 **b**	8.18 **b**	8.95 **b**	11.52 **a**	12.13 **a**	9.38 **b**	***	10.36	9.80	9.27	ns	ns
**2021**	**Kae**	0.34 **b**	0.36 **b**	0.40 **b**	0.65 **a**	0.67 **a**	0.34 **b**	***	0.57 **a**	0.42 **b**	0.39 **b**	**	ns
	**Que**	2.68 **b**	2.92 **b**	2.98 **b**	5.16 **a**	5.37 **a**	2.67 **b**	***	4.40 **a**	3.22 **b**	3.26 **b**	**	ns
	**Iso**	0.42 **b**	0.43 **b**	0.45 **b**	0.77 **a**	0.70 **a**	0.40 **b**	***	0.62 **a**	0.51 **ab**	0.46 **b**	**	ns
	**Di-OH**	3.09 **b**	3.34 **b**	3.44 **b**	5.93 **a**	6.07 **a**	3.07 **b**	***	5.02 **a**	3.74 **b**	3.71 **b**	**	ns
	**Myr**	0.24 **b**	2.66 **b**	2.86 **b**	4.00 **a**	4.10 **a**	2.49 **b**	***	3.53 **a**	3.04 **ab**	2.69 **b**	***	ns
	**Lar**	0.36	0.33	0.38	0.32	0.36	0.38	ns	0.40	0.34	0.32	ns	ns
	**Syr**	0.50 **ab**	0.49 **ab**	0.52 **a**	0.40 **c**	0.42 **bc**	0.51 **a**	**	0.49	0.47	0.46	ns	ns
	**Tri-OH**	3.27 **b**	3.48 **b**	3.76 **b**	4.72 **a**	4.89 **a**	3.39 **b**	***	4.42 **a**	3.84 **b**	3.50 **b**	***	ns
	**Total**	6.71 **b**	7.18 **b**	7.60 **b**	11.30 **a**	11.63 **a**	6.70 **b**	***	10.00 **a**	7.99 **b**	7.60**b**	**	ns

a Analysis of variance to compare data (p value indicated); Letters within columns indicate significant mean separation according to Tukey’s HSD test at p value< 0.05, where “*”: p value< 0.05; “**”: p value< 0.001, “***”: p value< 0.0001.

b VSP, vertical shoot positioned; VSP 60, vertical shoot positioned 60°; VSP 80, vertical shoot positioned 80°; SH, single high wire; HQ, high quadrilateral; Myr, myricetins; Que, quercetins; Kae, kaempferols; Lar, laricetins; Iso, isorhamnetin; Syr, syringetins; Tri-OH, tri-hydroxylated flavonols; Di-OH, di-hydroxylated flavonols; ET_c_, crop evapotranspiration; ns, not significant.

c All compounds were expressed in the unit of 10^-2^ mg per g of berry fresh weight.

### Flavonols and their correlations with canopy crown porosity and leaf area

**Figure 5:**
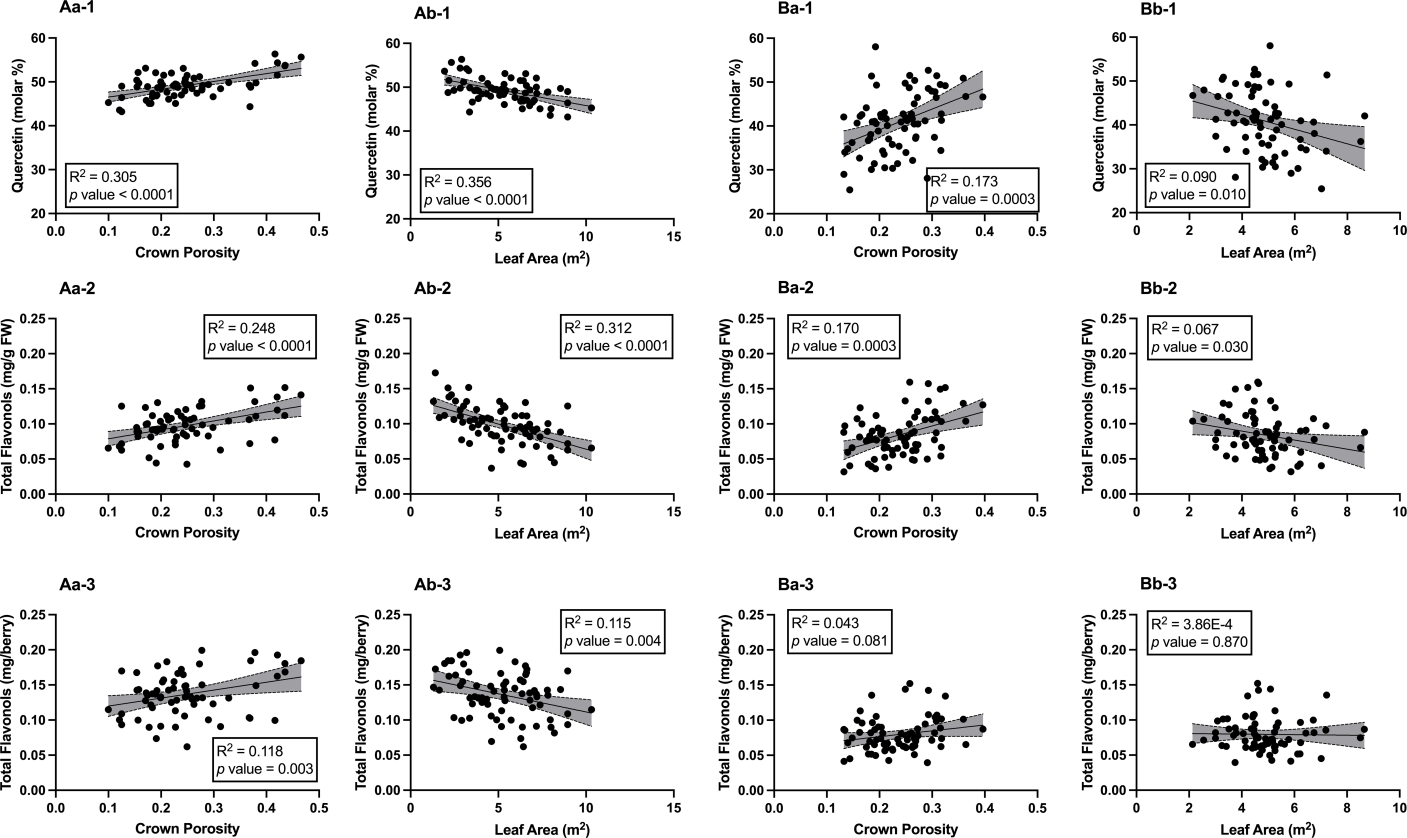
Relationships between Canopy Architecture and % molar Quercetin, Total Flavonol Concentration, and Total Flavonol Content in Berry Skins of ‘Cabernet Sauvignon’ in Oakville, CA, USA in 2020 **(A)** 2020 and **(B)** 2021; **(a)** correlations with crown porosity, **(b)** correlations with leaf areas; (1) quercetin (molar %), (2) total flavonols (mg/g FW), (3) total flavonols (mg/berry).Grey shade areas indicate 95% confidence intervals, and correlation values were expressed in R^2^ and *p* values. FW, berry fresh weight.

The relationships between berry skin flavonol concentrations and canopy architecture were investigated in both seasons (**Figure 5**). In 2020, crown porosity had positive and significant correlations with quercetin (molar %, R^2^ = 0.383, *p*< 0.0001, **Figure 5** **Aa-1**), total flavonol concentration (mg per g of berry fresh weight (FW), R^2^ = 0.248, *p*< 0.0001, **Figure 5** **Aa-2**), and total flavonol concentration (mg per berry, R^2^ = 0.118, *p* = 0.003, **Figure 5** **Aa-3**). Leaf area was also correlated with these variables, but the correlations were negative with quercetin (R^2^ = 0.356, *p*< 0.0001, **Figure 5** **Ab-1**), total flavonol concentration (R^2^ = 0.312, *p*< 0.0001, **Figure 5** **Ab-2**, and total flavonol concentration (R^2^ = 0.115, *p* = 0.004, **Figure 5** **Ab-3**). In 2021, the correlations were similar but not as significant as 2020. Crown porosity still had significant and positive relationships with quercetin (R^2^ = 0.173, *p* = 0.0003, **Figure 5** **Ba-1**) and total flavonols concentration (R^2^ = 0.170, *p* = 0.0003, **Figure 5** **Ba-2**). However, the relationship between crown porosity and total flavonol concentration (R^2^ = 0.043, *p* = 0.081, **Figure 5** **Ba-3**) did not persist, as was observed in 2020. The relationships between leaf area and quercetin (molar %) and total flavonol concentration were significant, although not as strong (R^2^ = 0.090, *p* = 0.010 and R^2^ = 0.067, *p* = 0.030, respectively). Leaf areas were negatively correlated with these two variables (**Figure 5** **Bb-1 and Bb-2**). The significant correlation between leaf area and total flavonol concentration did not hold in 2020 as compared to 2020 (R^2^ = 3.86E-04, *p* = 0.870, **Figure 5** **Bb-3**). It was evident that when crown porosity was greater, there was greater flavonol accumulation as well greater molar percentage of quercetin.

## Discussion

### Grapevine physiology was affected by canopy architecture and grapevine water status

A trellis system selected in grapevine vineyard is usually aimed at optimizing canopy architecture to further maximize canopy photosynthetic activity and improve canopy microclimate, which can yield desirable production and berry composition (Kliewer and Dokoozlian, 2005; Wessner and Kurtural, 2013; Sanchez-Rodriguez and Spósito, 2020). In historically cooler regions according to Winkler’s Index, VSP trellis system is widely used as it offers relatively higher compatibility with mechanization and is suitable for the regional production goals (Tardaguila et al., 2008). However, with the warming trend in air temperature getting more pronounced, VSPs have been showing greater chances of getting cluster overexposure, resulting in sunburnt berries with yield loss and color degradation (Martínez-Lüscher et al., 2017b; Torres et al., 2020a). Under our experimental conditions, HQ trellis system showed less leaf area and greater crown porosity than the other trellis systems in 2020 in accordance with previous studies, where split trellis designs might allow more solar radiation to penetrate the canopy interior (Wessner and Kurtural, 2013; Martínez-Lüscher et al., 2019a). Conversely, SH had similar leaf area but lower crown porosity in 2020. However, the differences in leaf areas and crown porosities were not as noticeable in 2021. This could be attributed to the fact that the HQ and SH might have still been filling up spaces with new growth compared to the VSPs, which might have already had relatively more established canopy architectures. In addition, the differences in leaf areas and crown porosities could be minimized by arid growing season in 2021, despite the supplemental irrigation applied to them, accounting for diminished leaf areas (mostly in VSPs) as shown in 2021 than 2020. Furthermore, precipitation received at the vineyard prior to bud break (Martínez-Lüscher et al., 2017a), as well as precipitation received immediately prior to flowering (Yu et al., 2021a) in semi-arid regions were deemed key determinants of canopy response for latter parts of the growing season.

In this study, yield per vine was not constantly determined by the trellis systems in both years, although similar bud densities at pruning were achieved. Furthermore, more leaf area did not account for more yield at harvest, despite it was well established that a sufficient leaf area would support fruit development (Martínez-Lüscher and Kurtural, 2021), and in contrast with some previous studies (Kliewer and Dokoozlian, 2005; Herrera et al., 2015). This could be attributed to not only the total amount leaf area but also how the leaves were distributed within the canopy. Commonly, HQ would have more open space to distribute more exposed and photosynthetically active leaves to the sunlight to optimize production (Bettiga et al., 2003; Brillante et al., 2018).

Previous studies have shown that greater leaf area can also contribute to higher TSS accumulation (Parker et al., 2015; Martínez-Lüscher and Kurtural, 2021), which was not observed in this study. On the contrary, more leaf area resulted in less TSS accumulation. This might be explained by the fact that the leaf area to fruit ratio, which represented the source-sink balance within the grapevine, might have a greater influence on the berry TSS accumulation. In this study, even though no statistical differences were observed in leaf area to fruit ratio, SH in 2020 showed relatively higher values (not statistically significant) with higher TSS accumulated at harvest. A similar situation was observed during the second season, where VSP80 showed relatively higher leaf area to fruit ratio (not statistically significant) and subsequently higher TSS at harvest. When crown porosity was considered, higher porosity resulted in greater TSS accumulation in berries, which could be attributed to the higher potential of berry exposure to the hot environment, causing the berries to experience greater dehydration (Torres et al., 2017). This relationship was not observed in 2021, and it might be derived from the relatively higher crop load and lower leaf area to fruit ratio in 2021 compared to 2020, especially by SH and HQ (not statistically significant). SH and HQ did not have a similar source-sink balance as 2020, which lowered their capacity to translocate photosynthates into the berries, this might have been the reason why they had a more reduced TSS at harvest compared to the other trellis systems.

Regarding the applied water amounts, the results were clear and consistent, with increased water status in grapevines irrigated with higher water amounts, and consequently, greater berry weight, cluster weight, and yield. These results agreed with previous studies on the relationships between grapevine water status and yield components (Torres et al., 2021b; Torres et al., 2021c). However, leaf area and crown porosity were not affected by applied water amount treatments in 2020. This might have been resulted from the remarkably high air temperature at the experimental site, diminishing the grapevine vegetative growth despite the water compensation from irrigation (Greer and Weedon, 2016). Consequently, berry quality parameters were slightly affected by irrigation treatments, with only TSS being higher with greater water stress in the harvest of the first season due to berry dehydration (Torres et al., 2017) and potential promotion in sugar accumulation (Zarrouk et al., 2016).

### Influences of trellis systems and grapevine water status on berry anthocyanins and flavonols

There were two flavonoid classes monitored in this study, anthocyanins and flavonols. They are highly sensitive towards environmental conditions (Martínez-Lüscher et al., 2014; de Rosas et al., 2017; Arrizabalaga-Arriazu et al., 2020; Yu et al., 2020). This study evidenced that SH increased berry skin anthocyanin and flavonol concentrations compared to the other trellis systems over the two seasons. SH might have had more advancement in berry development due to more efficient leaf area to fruit ratio achieved in this trellis system (Torres et al., 2017; Gambetta and Kurtural, 2021). Furthermore, the crown porosity of SH was ranging from 0.20 to 0.30, a window of inferred solar radiation exposure identified in previous works (Torres et al., 2020) for `Cabernet Sauvignon´. As for VSPs, anthocyanin degradation was unlikely to be the reason why VSPs had lower anthocyanin concentration since the greater leaf area could have provided berries some degree of protection from receiving excessive solar radiation (Torres et al., 2020). This can be confirmed by the fact that TSS and berry skin anthocyanin concentration were still synchronized in 2020. However, there was a decoupling of TSS and berry skin anthocyanin concentration in 2021, where the VSPs had higher TSS but lower skin anthocyanin content. Unlike the first season, the leaf area and canopy crown porosity showed no difference among the trellises, but the effective leaf area that can provide protection against excessive solar radiation might differ between SH and HQ from the other trellis systems. Hence, even with similar leaf areas, the VSPs still exposed clusters to the environmental stresses, which promoted TSS accumulation due to dehydration but greater anthocyanin degradation, similar to what was observed in previous studies (Martínez-Lüscher et al., 2019b; Yu et al., 2020). Although the TSS levels in this study were not at the level for reaching the tipping point of anthocyanin degradation as previously reported (approximately 24-25°Brix), compared to the SH and HQ with greater height from the vineyard floor, the VSPs might have been more easily affected by the solar radiation and heat reflected from soil surface, causing hotter and drier canopy microclimate and inevitably lead to greater anthocyanin degradation (Martínez-Lüscher et al., 2019a; reviewed by van Leeuwen et al., 2019). Additionally, some previous studies have shown negative relationships between yield and berry composition (Uriarte et al., 2016). Similar observations in this study might be due to source organs (leaves) of the VSPs were not distributed widely enough to be as efficient as those of the SH and HQ, resulting in lower photosynthetic capacity in their canopies, which further reduced the translocation of photosynthates flowing into berries to promote TSS accumulation and flavonoid biosynthesis.

As for flavonols, previous studies have shown that flavonols are very sensitive to solar radiation, especially UV radiation, where more light will often increase flavonol concentration in berry skins (Martínez-Lüscher et al., 2014; Torres et al., 2022). The results from this work corroborated previous observations that less leaf area with more crown porosity would increase solar radiation inside the canopy, and further increase flavonol concentrations in berry skins (Martínez-Lüscher et al., 2019a; Torres et al., 2021a). Additionally, SH and HQ showed greater concentrations in di-hydroxylated flavonols (quercetins and isorhamnetins) as well as some tri-hydroxylated flavonol (myricetins) derivatives.

Water deficits, achieved by manipulating applied water amounts through irrigation, can significantly improve flavonoid concentrations in grape berries (Torres et al., 2021d; Torres et al., 2021c). Similar results were observed in our findings as well, where 25% ET_c_ was able to increase anthocyanin and flavonol concentrations in grape berries. One previous study at the same experimental site showed that 25% ET_c_ could potentially increase the possibility for flavonoid degradation and decrease the wine antioxidant capacity (Torres et al., 2022). However, we did not see such effects in this study. This might be because berry sugar accumulation was not affected among the three applied water amounts, and the overall TSS levels did not exceed the tipping point (~25°Brix). It was repeatedly been noticed that, beyond this TSS level, skin anthocyanins and even flavonols would start to significantly degrade in a hot climate (Yu et al., 2020; Gambetta and Kurtural, 2021). Hence, in our study, all the treatments might have ended up having similar advancements in berry flavonoid accumulation because of the similar levels of TSS without any promoted accumulation or degradation among the three irrigation strategies (Ferri et al., 2011), but 25% ET_c_ was able to decrease berry weights, which resulted in higher concentrations in anthocyanins and flavonols.

### Flavonols as an indicator of canopy architecture determined by solar radiation

Positive relationships between flavonols and solar radiation, especially UV-B, have been consistently observed in previous research, clearly indicating that more solar radiation penetrating into the canopy interior promotes flavonol concentration in berry skins (Koyama et al., 2012; Martínez-Lüscher et al., 2014). Further, flavonol content and derivative proportions exhibited strong relationships with solar radiation (Martínez-Lüscher et al., 2019a), which was confirmed in this study, where quercetin proportion and both total flavonol concentration correlated strongly with leaf area and crown porosity especially with VSP types.

When the high air temperature or drought conditions became extreme, flavonoids in berry skins started to degrade (Martínez-Lüscher et al., 2017b). For all six trellis systems in 2020, the relationships between flavonols and canopy architecture were strong. These relationships between leaf area/crown porosity and flavonols can provide a feasible way of assessing canopy architecture in terms of the canopy’s contribution towards berry composition and *vice versa*. This approach is not limited only to red cultivars and can also be applied to white cultivars since flavonols are still synthesized in their skin tissues (Pérez-Navarro et al., 2021). Also, for quercetin specifically, it is the most abundant flavonol derivative in grape berry skins. Hence, the compound would be unchallenging to isolate and extract, offering an easy assessment of the effects of solar radiation on berry flavonol profiles. Interestingly, in this study, the VSPs did not result in higher quercetin or total flavonol concentrations, indicating that these trellis systems might not be suitable for accumulating or maintaining flavonoids in berry skins in a hot climate regardless of TSS levels compared to other trellis systems. Although the relationships between canopy architecture and flavonols were strong in this study and align with previous reports, the influence of canopy structure imposed by trellis system on berry chemical development needs more investigation to understand the contributions of trellis systems to canopy architecture and canopy microclimate.

## Conclusion

As growing season temperatures continue to rise in viticultural regions, grape growers are looking for ways to adapt to maintain consistent production volume and quality. However, legislative pressure on grape growers harnessing their ability to extract ground water for irrigation purposes will limit this adaptation. Overall, this study provided evidence of how different trellis systems combined with irrigation strategies affected grapevine physiological development and berry chemical profiles. Our results indicated that SH and HQ trellis systems could enhance the efficiency of grapevine canopy in promoting TSS accumulation and yield as well as higher capacity for flavonol and anthocyanin accumulation in berry skins with less chemical degradation compared to the traditional VSPs. Additionally, we purposely aimed to study the relationships between flavonols and canopy architecture. We observed strong correlations between molar % quercetin, and total flavonol concentration and content with leaf area and canopy porosity, indicating that berry skin flavonols can be feasible indicators for canopy architecture to register berry development in response to solar radiation.

## Data availability statement

The raw data supporting the conclusions of this article will be made available by the authors, without undue reservation.

## Author contributions

SK conceptualized and designed the trial. RY, NT, JG, LM, MZ, JT, and SK executed the trial, curated the data. RY and NT wrote the first version of the manuscript. GG and SK revised the manuscript. All authors contributed to the article and approved the submitted version.

## Funding

UC Davis Library has provided partial funding to defray publication costs.

## Conflict of interest

The authors declare that the research was conducted in the absence of any commercial or financial relationships that could be construed as a potential conflict of interest.

## Publisher’s note

All claims expressed in this article are solely those of the authors and do not necessarily represent those of their affiliated organizations, or those of the publisher, the editors and the reviewers. Any product that may be evaluated in this article, or claim that may be made by its manufacturer, is not guaranteed or endorsed by the publisher.
